# Headspace Gas Chromatography-Mass Spectrometry for Volatile Components Analysis in *Ipomoea Cairica* (L.) *Sweet* Leaves: Natural Deep Eutectic Solvents as Green Extraction and Dilution Matrix

**DOI:** 10.3390/foods8060205

**Published:** 2019-06-11

**Authors:** Wei Zhang, Xianrui Liang

**Affiliations:** Collaborative Innovation Center of Yangtze River Delta Region Green Pharmaceuticals, College of Pharmaceutical Sciences, Zhejiang University of Technology, Hangzhou 310014, China; 15958100694@163.com

**Keywords:** *Ipomoea cairica* (L.) *Sweet* leaves, natural deep eutectic solvents, static headspace gas chromatography-mass spectrometry, volatile components

## Abstract

In this study, natural deep eutectic solvents (NADESs) were used as both the extraction and dilution matrix in static headspace gas chromatography-mass spectrometry (SHS-GC-MS) for the analysis of volatile components in *Ipomoea cairica* (L). *Sweet* (*ICS*) leaves. Six NADESs were prepared and the NADESs composed of choline chloride and glucose with a 1:1 molar ratio containing 15% water were preferred due to the better peak responses. A total of 77 volatiles in *ICS* leaves were detected and tentatively identified by mass spectral matching with the US National Institute of Standards and Technology (NIST, 2014) Mass Spectral Library and the retention index-assisted qualitative method. These 77 volatile components were mainly terpenoids, aromatics, and aliphatics. Among them, β-elemene, β-caryophyllene, α-humulene, and 2, 4-di-tert-butylphenol were found to be the main components. This investigation verified that the use of NADESs is an efficient green extraction and dilution matrix of the SHS-GC-MS method for direct volatile component analysis of plant materials without extra extraction work.

## 1. Introduction

Plant volatiles are a series of low molecular weight lipophilic compounds derived from different biosynthetic pathways during plant growth [1]. The volatile components in the plant materials are usually produced for defensive strategies against biotic and abiotic stresses, contributing to the various antimicrobial and antioxidative properties [2,3,4]. These volatile components are often extracted as essential oils, which have a long history of applications in the pharmaceutical, agricultural, and cosmetic industries [5,6]. The bioactivities of essential oils are often greatly influenced by the chemical composition, which makes the chemical characterization of volatile components an important work [7,8,9]. Hence, there is still a lot of structural identification and bioactivity research work to do for the development and application of diverse natural plant materials.

*Ipomoea cairica (L.) Sweet* (*ICS*), belonging to the perennial herbaceous vines of the genus, sweet potato of convolvulacea, with a wide distribution nearly all over the world, is used worldwide as folk medicine [10,11,12]. Pharmacological activity research has also revealed that *ICS* has cytotoxic, larvicidal, anti-inflammatory, and antinociceptive activities [13,14,15,16,17,18,19]. The various bioactivities are relevant to the diverse components in *ICS* [20]. For example, larvicidal and antinociceptive activities have been demonstrated to be related to the volatile components in *ICS* [20]. However, the volatile components’ profile of *ICS* has not been well explored.

Static headspace gas chromatography mass spectrometry (SHS-GC-MS) is a powerful and efficient way to profile the composition of complex volatile components due to its effectiveness in simultaneous chromatographic separation and structural identification [21,22]. Compared to direct injection, SHS sampling is clean and convenient as a gas extraction technique for GC-MS analysis. Although SHS has been used as a mature technique for decades, the method’s optimization work has never been terminated. One of the important works is exploring the superior dilution matrix. Except for the traditionally used dilution matrix of dimethyl sulfoxide (DMSO), dimethylacetamide (DMA), and water, many other high boiling solvents, such as paraffin and ionic liquids, have also been used as SHS matrix medium [23,24,25,26]. Recently, we successfully introduced another new type of green solvent natural deep eutectic solvents (NADESs) into SHS for the analysis of residue solvents in pharmaceuticals [27]. The NADESs exhibited many superiorities over the traditionally used matrix, with easy preparation, very low price and toxicity, biodegradability, and, most important of all, good sensitivity [28,29,30,31]. NADESs have also been demonstrated to be good solvents for the simultaneous extraction of hydrophilic and hydrophobic compounds from plant materials [32].

To further explore the application of NADESs as a green and friendly dilution matrix medium, volatile compounds in *ICS* leaves were analyzed by the SHS-GC-MS method in this study. The NADESs was used as both the extraction and dilution media. Six choline chloride based NADESs were prepared. The key SHS-GC parameters were optimized. Based on the optimized conditions, the structures of volatile components in *ICS* leaves were illustrated. The results of this study verified the feasibility of NADESs as a headspace extraction and dilution matrix in the analysis of volatile components from plant materials, and also provided useful information for the development and application of *ICS* in foods and medicals.

## 2. Materials and Methods

### 2.1. Regents and Materials

Leaves of *ICS* were collected from Meizhou, Guangdong and identified by Aichun Zhou in Zhejiang Agriculture and Forestry University. It was stored at −4 °C until use. Levulinic acid (purity ≥ 99.0%) were purchased from Aladdin Biochemical Co., Ltd. (Shanghai, China). Glucose (purity ≥ 98.0%), citric acid (purity ≥ 99.5%), maltose (purity ≥ 98.0%), fructose (purity ≥ 98.0%), and 1,4-butanediol (purity ≥ 99.0%) were purchased from Sinopharm Chemical Reagent Co., Ltd. (Beijing, China). Choline chloride (purity ≥ 98.0%) were purchased from Yuanye Biological Co., Ltd. (Shanghai, China). The reagents and chemicals used above were all of analytical grade. The HPLC-grade n-hexane (purity ≥ 95.0%) was purchased from TEDIA (Cincinnati, OH, USA). The C_7_–C_40_ *n*-alkane saturated alkane mixture (1000 mg/L) used as reference standards were purchased from Sigma-Aldrich Co., Ltd. (Shanghai, China).

### 2.2. Preparation of NADESs

NADESs were prepared by heating method [31]. The HBAs with different HBDs in different molar ratios were mixed in a conical flask with a cover and magnetically stirred at 85 °C by a water bath until a homogeneous liquid formed. Standing at room temperature, NADES was completed if no solid precipitation occurred. The NADES-1,2,5 were composed of choline chloride and citric acid, glucose, and maltose with a 1:1 molar ratio. The NADES-3, 4 were composed of choline chloride and levulinic acid, 1,4-butanediol with 1:2 molar ratio. The NADES-6 was composed of choline chloride and fructose with a 2:1 molar ratio.

### 2.3. Preparation of ICS Leaves Powder

The *ICS* leaves were pulverized and sifted by an 80 mesh sieve. The powder was further triturated on a Retsch PM200 planetary ball mill (Retsch Inc., Haan, Germany) to obtain smaller particle size powder under optimal conditions. The optimized ball milling conditions were: The ball to powder weight ratio was 1:30, rotating speed was 300 rpm, and rotating time was 25 min.

### 2.4. SHS-GC-MS Analysis Conditions

The SHS-GC-MS analysis was performed in an Agilent 7890B-5977A gas chromatograph-mass spectrometer equipped with an Agilent 7697A Headspace auto-sampler (Agilent Technologies Inc., Santa Clara, CA, USA). A J&W capillary column HP-5 MS UI of 30 m × 0.250 mm with 0.25 μm film (Agilent Technologies Inc.) was used for the separation. The *ICS* samples in a 20 mL headspace vial were heated at an equilibrium temperature of 130 °C for 40 min, and the gas phase were injected into the GC-MS for analysis. The injection time was 1.0 min. A low shaker mode of the headspace vial was applied during sample heating.

GC parameters were as follows: The carrier gas and make-up gas were high pure helium and nitrogen, respectively. The carrier gas (helium) was set at a flow rate of 1.0 mL·min^−1^. The inlet temperature was 200 °C with a split ratio of 10:1 and the pressure was 11.6 psi. The column oven temperature was initially set at 60 °C for 5 min, and then ramped to 200 °C at 5 °C·min^−1^ for 5 min, and after that, it was warmed up to 300 °C at 10 °C·min^−1^.

MS parameters were as follows: Data were acquired in the electron impact (EI) mode, using the full scan mode from *m/z* 30 to 600 at 1562 amu/s. The ion source temperature and quadrupole temperature were 230 and 150 °C, respectively. The identification of volatile compounds was based on a comparison of their GC retention time and mass spectra with the retention index of n-alkane saturated alkanes and the reference spectra from the US National Institute of Standards and Technology (NIST, 2014). The values were the mean of three replicates of each sample. Data were analyzed by using Agilent MassHunter Analysis.

### 2.5. Preparation of ICS Samples for SHS-GC-MS Analysis

About 1 g of *ICS* powder was accurately weighed and added into a 20 mL headspace vial by adding 5 mL NADES, mixed and sealed for SHS-GC-MS analysis. For analysis work, all the NADESs were added to 15% water in a weight ratio, respectively, except for the optimization experiments of the water content in NADESs. The solid–liquid ratio in the headspace vial was 1:5 (g:mL) for all the experiments except the optimization experiments of the solid–liquid ratio. The other experiment conditions were the same as that listed in Section 2.4. Each sample was determined in triplicate.

### 2.6. Determination of Retention Index

A 500 μL pipette of C_7_–C_40_ n-alkane saturated alkane mixture was placed into a 10 mL volumetric flask and diluted to the volume with n-hexane to make a concentration of 50 μL/mL solution. Then, 3 mL of alkane solution was transferred to a 20 mL headspace vial for SHS-GC-MS analysis. The SHS-GC-MS conditions were the same as that in Section 2.4. The sample was analyzed in triplicate.

The formula used for calculating the retention index was as follows:

(1) $$RI=100Z+100\frac{TR(x)-TR(z)}{TR(z+1)-TR(z)},$$

where *TR*(*x*), *TR*(*z*), and *TR*(*z* + 1) represent the retention temperature of the component, the n-alkane with carbon number at *z* and *z* + 1, respectively, and *TR*(*z*) < *TR*(*x*) < *TR*(*z* + 1). The retention temperature was obtained according to the temperature programming.

## 3. Results and Discussions

### 3.1. Optimization of SHS Parameters

The equilibrium time and temperature were the key operating parameters affecting the SHS efficiency. The equilibrium temperature often depends on the dilution matrix used in SHS. To obtain the optimized equilibrium temperature, different temperatures of 100, 110, 120, 130, and 140 °C were carried out in NADES-2 containing 15% water for SHS-GC-MS analysis. As shown in Figure S1a, with the increase of the temperature, the number of peaks detected in the ICS samples increased. When the equilibrium temperature was raised to 130 °C, greater peak numbers with a better response were observed. Similar results were obtained at 140 °C, but the peak numbers were somewhat decreased. Although the high temperature was beneficial for increasing the peak response of the volatile compounds, the excessive high temperatures could result in the degradation of thermally unstable compounds. As a result, 130 °C was selected as the preferred equilibrium temperature.

On the other hand, the appropriate equilibrium time also played an important role for achieving the gas–liquid balance. Similar to the above operation, the equilibrium time at 20, 30, 40, 50, and 60 min were evaluated in NADES-2 containing 15% water with the other experiment conditions the same as that listed in Section 2.5. As shown in Figure S1b, the number of peaks detected at 40 min were more than those at other equilibrium times and 40 min was chosen to be the preferred equilibrium time.

### 3.2. Selection of NADESs for SHS-GC-MS Analysis

Six NADESs were prepared, including choline chloride-citric acid, in a 1:1 molar ratio (NADES-1), choline chloride-glucose in a 1:1 molar ratio (NADES-2), choline chloride-1, 4-butanediol in a 1:2 molar ratio (NADES-3), choline chloride-levulinic acid in a 1:2 molar ratio (NADES-4), choline chloride-fructose in a 1:1 molar ratio (NADES-5), and choline chloride-maltose (NADES-6). Transparent solutions were obtained and no precipitation were observed during storage at room temperature. All these NADESs were added to 15% water in a weight ratio before application for further analysis.

To verify the feasibility of NADESs as an SHS dilution matrix, the comparison of the total ion chromatograms of ICS samples between the ICS essential oil, which was prepared by steam distillation, and the ICS powder in NADES-2 containing 15% water were preliminarily performed as shown in Figure S2. These two samples were detected in the same experiment conditions except different injection methods were used. The ICS essential oil was detected by the direct GC-MS method while the ICS powder was put into a headspace vial with the addition of NADES-2 containing 15% water as the dilution matrix and determined by the SHS-GC-MS method. The results showed that the relatively higher responses with more components were detected from the latter than the former, especially the low boiling point components, which were probably lost by the extraction process of the essential oil. It was reported that NADESs can be used to dissolve cellulose and lignin which facilitate the release of compounds from plant tissues [33,34]. As a dilution matrix in SHS, the NADESs used in this work also exhibited good dissolving or extracting properties for the volatile components in *ICS* leaves.

**Figure 1:**
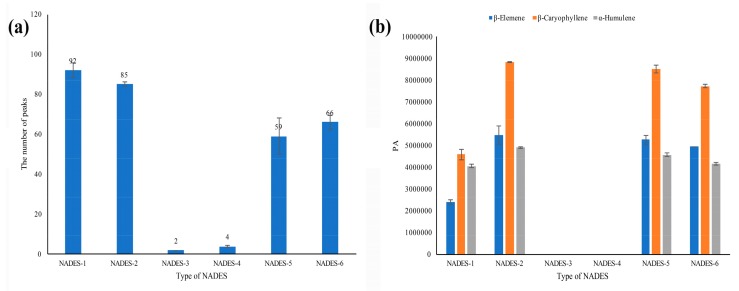
Comparison of peak responses of *Ipomoea cairica (L.) Sweet* (*ICS*) samples in different natural deep eutectic solvents (NADESs) containing 15% water: (**a**) the number of peaks; (**b**) the peak areas of three main components.

However, for the six NADESs prepared in this study, they exhibited different analysis characteristics as shown in Figure 1. It was found that three sesquiterpenes, including β-elemene, β-caryophyllene, and α-humulene, were the main volatile components in the *ICS* leaves. The peak areas of these three main compounds and the number of peaks detected were used as one of the key parameters for further analysis of the optimal conditions. A few components were detected in both NADES-3 and NADES-4. More components were observed in NADES-1 and 2 than in NADES-5 and 6. Although a little bit more components were observed in NADES-1, the higher peak areas of the three main compounds were obtained in NADES-2, and NADES-2 was selected as the optimized dilution matrix for further investigation.

### 3.3. Effects of the Solid–Liquid Ratio and Water Content of NADESs on SHS Efficiency

**Figure 2:**
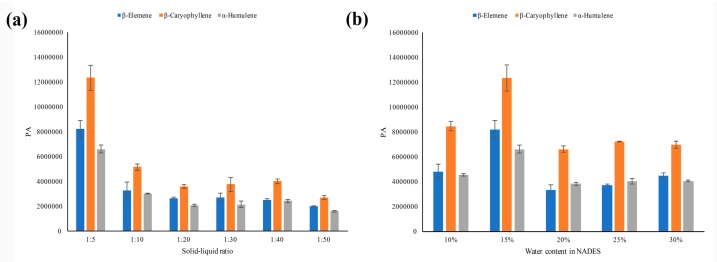
The peak areas of the three main components in *ICS* leaves: (**a**) in different solid–liquid ratios; (**b**) NADES-2 containing different amount of water.

The peak responses of compounds or the headspace efficiency were greatly affected by the concentration ratio between the analyte in the dilution matrix and the gas phase in the headspace vial [35]. It was reasonable that a large amount of dilution matrix would decrease the equilibrium concentration of the analyte in the gas phase of the headspace vial and hence decrease the headspace efficiency. To explore the suitable amount of NADESs used in headspace vials, different solid–liquid ratios (ratios of the ICS powder weight and the NADES volume) ranging from 1:5 to 1:50 were investigated. Since the peak numbers were similar, the peak areas of the three main components were used as the key parameters for comparison and the results are shown in Figure 2a. With an increasing of the solid–liquid ratios, the peak areas of three main components decreased. As a result, the solid-liquid ratio at 1:5 was preferred.

Although NADES has been demonstrated to have many superiorities and has a wide application as a new type of solvent, its high viscosity makes its application somewhat inconvenient [36,37]. The viscosity of NADESs could be largely decreased by adding water. In this study, the NADES-2 with the addition of 10%, 15%, 20%, 25%, and 30% (wt%) water were evaluated for the SHS efficiency. Figure 2b showed that the peak areas of the three main components increased slightly by adding 10% to 15% of water. However, when the amount of water was over 15%, the peak areas decreased. It may be attributed to the fact that the hydrogen bonding network in NADES is weakened by adding a large amount of water [38]. As a result, the NADES-2 with 15% water content was the better choice.

### 3.4. Volatile Components’ Characterization by SHS-GC-MS

**Figure 3:**
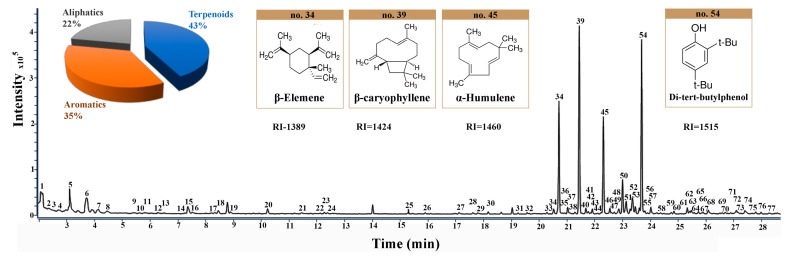
The total ion chromatography (TIC) of *ICS* leaves in NADES-2 containing 15% water and the molecular structures of main volatile constituents (No. 34, 39, 45, and 54).

The total ion chromatography (TIC) under the optimized conditions is shown in Figure 3. A total of 77 volatile compounds in the *ICS* leaves were tentatively illustrated by matching the mass spectra with the NIST14 Mass Spectral Library and the NIST MS Search 2.2 for the reference retention index. The mass spectra matching was affected by many factors, such as the reference spectral library, spectral similarity measurement, and weight factor [39,40]. In addition, mass spectral matching cannot distinguish isomers from each other. For natural products, isomers were the common phenomena and showed very similar mass spectra. To differentiate these isomers, the retention index provides a better choice due to the different retention index of the isomers [41,42]. In this work, the retention index of the components was calculated and used for the identification of compounds in *ICS* leaves as listed in Table 1.

#### 3.4.1. Identification of Terpenoids

Terpenoids were the main volatile components in the *ICS* leaves. Compounds **23**, **31**, **32**, **33**, **34**, **36**, **39**, **40**, **43**, **44**, **45**, **46**, **49**, **50**, **53**, **56**, **58**, **59**, **60**, **61**, **62**, **63**, **65**, **67,** and **75** were identified as terpenoids. Among them, the β-caryophyllene (compound **39**), β-elemene (compound **34**), and α-humulene (compound **45**) were observed to be the main components. Most terpenoids have abundant pharmacological activities. They are used for analgesic and itching, and have antiseptic and bactericidal effects. β-Elemene is useful for killing tumor cells and conventional doses have no effect on normal cells [43]. β-Caryophyllene [44,45] is widely used as a spice in cosmetics and food additives. Pharmacological studies showed that β-caryophyllene has local anesthetic, anti-inflammatory, insect repellent, anti-anxiety and anti-depression effects. β-Caryophyllene is also used in antitussive and expectorant drugs. Compound **34** was used as an example to elucidate the identification work of these terpenoids. The mass spectrum of compound **34** showed the molecular ion at *m/z* 204.2 and the fragment ions at *m/z* 189.2, 175.1, 161.1, 147.1, 133.1, 121.1, 107.1, 93.1, 81.1, 68.1, 55.1, 41.1, and 32.0, respectively. The mass spectral matching result showed that β-elemene had a high matching score of 97. The retention index of compound **34** was 1389, which was similar to the reference retention index 1398 of β-elemene. As a result, compound **34** was identified as β-elemene.

#### 3.4.2. Identification of Aromatics

Compounds **14**, **15**, **19**, **20**, **24**, **37**, **41**, **42**, **48**, **51**, **52**, **54**, **55**, **57**, **64**, **69**, **70**, **71**, **72**, **74**, and **76** were identified as aromatic compounds. The high content of di-tert-butylphenol (compound **54**) was found in the *ICS* leaves, which is reported to have antifungal and antioxidant activities [46]. Compound **54** was used as an example to elucidate the identification work of these aromatics. The mass spectrum of compound **54** showed that the molecular ion at *m/z* 206.2 and the fragment ions were *m/z* 191.1, 175.1, 163.1, 147.1, 128.1, 107.0, 91.0, 74.0, 66.0, 57.1, 51.0, 40.0, and 32.0, respectively. The mass spectral matching result showed that 2,4-di-tert-butylphenol had a high matching score of 92. The retention index of compound **54** was 1515, which was the similar to the reference retention index 1519. The compound **54** was identified as 2,4-di-tert-butylphenol.

#### 3.4.3. Identification of Aliphatics

Compounds **1–13, 17–18, 21, 22, 25–30, 35, 38, 47, 66, 68, 73**, and **77** were identified as aliphatic compounds. The contents of aliphatic compounds were relatively low. Compound **6** was used as an example to elucidate the identification work of these aromatics. The mass spectrum of compound **6** showed that the molecular ion at *m/z* 96.0 and the fragment ions were *m/z* 87.0, 73.0, 67.0, 58.1, 51.0, 44.0, 39.0, and 32.0, respectively. The mass spectral matching result showed that 3-furaldehyde had a high matching score of 94. The retention index of compound **6** was 829, which was similar to the reference retention index 832 of 3-furaldehyde. As a result, the compound **6** was identified as 3-furaldehyde.

## 4. Conclusions

In conclusion, the green solvent NADESs as both an extraction and dilution matrix were successfully applied to the volatile components analysis in *ICS* leaves by the SHS-GC-MS method. Different kinds of NADESs exhibited different SHS efficiencies for the various volatile components. For the volatile components in *ICS* leaves, the NADES composed of choline chloride and glucose with a 1:1 molar ratio containing 15% water showed the preferred results. With the optimized analysis conditions, a total of 77 volatiles in *ICS* leaves were tentatively identified and assigned by mass spectral matching with the standard mass spectral library and the retention index-assisted qualitative method. The main contents in *ICS* leaves were β-elemene, β-caryophyllene, α-humulene, and 2,4-di-tert-butylphenol. Based on the results obtained in this study, NADESs were demonstrated to be the efficient green extraction and dilution matrix in the SHS-GC-MS method and could be directly used for volatile components analysis of plant materials without extra extraction work.

## Figures and Tables

**Table 1:** Compounds tentatively illustrated of *Ipomoea cairica Sweet* leaves detected by SHS-GC-MS.

No.	Components	RT	Formula	CAS	Score	RI	Contents * (%)
1	5-Methyl-5-hexen-2-ol	2.07	C_7_H_14_O	50551-88-7	70.73	692	3.65
2	2-Ethyl-furan	2.14	C_6_H_8_O	3208-16-0	77.37	708	1.41
3	1-(Methylencyclopropyl)-ethanol	2.36	C_6_H_10_O		70.89	725	0.14
4	4-Penten-1-ol	2.69	C_5_H_10_O	821-09-0	65.28	766	0.43
5	2, 4-Dimethyl-3-pentanol	3.10	C_7_H_16_O	600-36-2	80.18	803	3.75
6	3-Furaldehyde	3.72	C_5_H_4_O2	498-60-2	90.06	829	4.73
7	(E)-2-hexenal	4.15	C_6_H_10_O	6728-26-3	80.70	849	1.07
8	4-Methyl-2-penten-1-ol	4.46	C_6_H_12_O	5362-55-0	67.13	863	0.59
9	Heptanal	5.42	C_7_H_14_O	111-71-7	67.55	904	0.27
10	(+/-)-2-Amino-1-propanol	5.61	C_3_H_9_NO	2749-11-3	72.19	910	0.13
11	1-(2-Furanyl)-ethanone,	5.83	C_6_H_6_O_2_	1192-62-7	60.92	916	0.30
12	Butyrolactone	6.26	C_4_H_6_O_2_	96-48-0	63.77	923	0.21
13	(1R)-2, 6, 6-Trimethylbicyclo [3.1.1] hept-2-ene	6.50	C_10_H_16_	7785-70-8	54.28	936	0.14
14	1-Methyl-3-cyclohexen-1-ol	7.27	C_7_H_12_O	33061-16-4	53.39	959	0.12
15	Benzaldehyde	7.36	C_7_H_8_O	100-52-7	87.31	962	1.52
16	1-Methyl-pyrazole-4-carboxaldehyde	7.53	C_5_H_6_N_2_O	25016-11-9	73.89	966	0.41
17	2-Methyl-1-hepten-6-one	8.34	C_8_H_14_O	10408-15-8	69.68	991	0.19
18	2-Pentyl-furan	8.46	C_9_H_14_O	3777-69-3	77.14	994	0.57
19	1-Methyl-1H-pyrrole-2-carboxaldehyde	8.90	C_6_H_7_NO	1192-58-1	67.63	1004	0.31
20	Benzeneacetaldehyde	10.22	C_8_H_8_O	122-78-1	85.65	1023	0.81
21	2, 5-Furandicarboxaldehyde	11.44	C_6_H_4_O_3_	823-82-5	61.79	1042	0.19
22	Nonanal	12.27	C_9_H_18_O	124-19-6	74.41	1107	0.20
23	Levomenthol	12.38	C_10_H_20_O	2216-51-5	65.21	1110	0.16
24	Phenylethyl alcohol	12.57	C_8_H_10_O	60-12-8	73.38	1116	0.17
25	2, 6, 6-Trimethyl-1, 3-cyclohexadiene-1-carboxaldehyde	15.27	C_10_H_14_O	116-26-7	61.78	1202	0.10
26	2, 6, 6-Trimethyl-1-cyclohexene-1-carboxaldehyde	15.90	C_10_H_16_O	432-25-7	64.44	1204	0.16
27	4-(2, 6, 6-Trimethyl-1, 3-cyclohexadien-1-yl)-2-butanone	17.11	C_13_H_20_O	20483-36-7	66.03	1266	0.20
28	(1R, 2R, 5R, E)-7-Ethylidene-1, 2, 8, 8-tetramethylbicyclo [3.3.1] octane	17.61	C_14_H_14_	193695-14-6	54.09	1283	0.17
29	Tetrahydro-6-propyl-2H-pyran-2-one	17.79	C_8_H_14_O_2_	542-28-9	69.71	1289	0.19
30	2, 6, 10, 10-Tetramethyl-1-oxaspiro [4.5] dec-6-ene	18.17	C_13_H_22_O	36431-72-8	80.79	1302	0.25
31	(3R-trans)-4-Ethenyl-4-methyl-3-(1-methylethenyl)-1-(1-methylethyl)-cyclohexene	19.24	C_15_H_24_	20307-84-0	73.27	1342	0.14
32	trans-Calamenene	19.57	C_15_H_22_	73209-42-4	74.69	1355	0.14
33	α-Cubebene	20.28	C1_5_H_24_	17699-14-8	81.24	1381	0.17
34	β-Elemene	20.52	C_15_H_24_	515-13-9	97.06	1389	0.63
35	2, 4, 6-Trimethyl-decane	20.85	C_12_H_26_	2801-84-5	51.23	1402	0.11
36	δ-Selinene	20.92	C_15_H_24_	28624-23-9	71.98	1405	0.11
37	4-(Dimethylamine)-benzaldehyde	21.02	C_9_H_11_NO	100-10-7	90.17	1409	0.90
38	(E)-1-(2, 3, 6-trimethylphenyl) buta-1, 3-diene (TPB,1)	21.29	C_13_H_16_	1000357-25-7	58.90	1419	0.23
39	β-Caryophyllene	21.44	C_15_H_24_	87-44-5	98.21	1424	22.68
40	(+)-epi-Bicyclosesquiphellandrene	21.67	C_15_H_24_	54274-73-6	93.89	1435	0.75
41	[1aR-(1aα, 7aα, 7bα)]-1a, 2, 3, 5, 6, 7, 7a, 7b-Octahydro-1, 1, 7, 7a-tetramethyl-1H-cyclopropa [a] naphthalene	21.76	C_15_H_24_	17334-55-3	82.99	1439	0.32
42	(1S-cis)-1, 2, 3, 5, 6, 8a-Hexaahydro-4, 7-dimethyl-1-(1-methylethyl)-naphthalene	21.84	C_15_H_24_	483-76-1	75.42	1442	0.12
43	α-Guaiene	21.91	C_15_H_24_	3691-12-1	90.65	1444	0.58
44	cis-Calamenene	22.23	C_15_H_22_	72937-55-4	69.79	1457	0.18
45	α-Humulene	22.31	C_15_H_24_	6753-98-6	97.41	1460	11.23
46	Bicyclosesquiphellandrene	22.54	C_15_H_24_	54324-03-7	91.10	1469	0.61
47	2, 6-Bis (1, 1-dimethylethyl)-2, 5-cyclohexadiene-1, 4-dione	22.61	C_14_H_20_O_2_	719-22-2	91.04	1472	0.11
48	cis-Muurola-4(15), 5-diene	22.70	C_15_H_24_	157477-72-0	68.22	1476	0.14
49	(1α, 4aβ, 8aα)- (+/-)-1, 2, 4a, 5, 8, 8a-Hexahydro-4, 7-1-(1-methylethyl)-naphthalene	22.86	C_15_H_24_	5951-61-1	89.26	1482	0.75
50	β-Guaiene	23.00	C_15_H_24_	88-84-6	93.97	1487	4.09
51	[4aR-(4aα, 7α, 8aβ)]-Decahydro-4a-methyl-1-methylene-7-(1-methylethenyl)-naphthalene	23.13	C_15_H_24_	17066-67-0	87.43	1493	2.13
52	2-Isopropenyl-4a, 8-dimethyl-1, 2, 3, 4, 4a, 5, 6, 8a-octahydronaphthalene	23.35	C_15_H_24_	1000193-57-0	90.13	1501	2.78
53	[1S-(1α, 7α, 8aβ)]-1, 2, 3, 5, 6, 7, 8,8a-octahydro-1,4-dimethyl-7-(1-methylethenyl)-azulene	23.60	C_15_H_24_	3691-11-0	89.20	1512	0.70
54	Di-tert-butylphenol	23.69	C_14_H_22_O	96-76-4	93.23	1515	20.11
55	1, 2, 3, 4, 4a, 5, 6, 8a-Octahydro-7-methyl-4-methylene-naphthalene	23.80	C_15_H_24_	39029-41-9	76.36	1520	0.37
56	Selina-3, 7(11)-diene	23.90	C_15_H_24_	6813-21-4	54.29	1525	0.12
57	(1S-cis)-1, 2, 3, 5, 6, 8a-Hexahydro-4, 7-dimethyl-1-(1-methylethyl)-naphthalene	24.02	C_15_H_24_	483-76-1	90.16	1530	0.90
58	Epizonarene	24.37	C_15_H_24_	41702-63-0	63.48	1544	0.16
59	Caryophyllene oxide	24.74	C_15_H_24_O	1139-30-6	59.71	1560	0.18
60	Patchoulene	24.84	C_15_H_26_	25491-20-7	85.19	1565	0.34
61	Dehydro-aromadendrene	25.32	C_15_H_22_		85.63	1585	0.78
62	β-Vatirenene	25.41	C_15_H_22_	27840-40-0	72.71	1588	0.35
63	Aristol-1(10)-en-9-ol	25.47	C_15_H_24_O	1372763-27-3	81.55	1591	0.54
64	(8R, 8aS)-8, 8a-Dimethyl-2-(propan-2-ylidene)-1, 2, 3, 7, 8, 8a-hexahydronaphthalene	25.57	C_15_H_22_	27840-40-0	69.23	1595	0.15
65	Salvial-4(14)-en-1-one	25.73	C_15_H_24_O	73809-82-2	66.55	1602	0.31
66	1, 3-Bis-(2-cyclopropyl,2-methylcyclopropyl)-but-2-en-1-one	25.85	C_18_H_26_O		72.68	1607	0.11
67	[3R-(3α, 3aβ, 7β, 8aα)]-2, 3, 4, 7, 8, 8a-Hexahydro-3, 6, 8, 8-tetramethyl-1H-3a, 7-methanoazulene	25.90	C_15_H_24_	469-61-4	74.34	1610	0.19
68	(+)-Epi-, β-santalyl acetate	26.09	C_17_H_26_O_2_	41414-75-9	75.95	1617	0.64
69	(1S, 7S, 8aR)-1, 8a-Dimethyl-7-(prop-1-en-2-yl)-1, 2, 3, 7, 8, 8a-hexahydronaphthalene	26.60	C_15_H_22_	190327-38-9	61.30	1640	0.11
70	4a, 5-Dimethyl-3-(prop-1-en-2-yl)-1, 2, 3, 4, 4a, 5, 6, 7-octahydronaphthalen-1-ol	26.69	C_15_H_24_O	61847-19-6	67.23	1645	0.19
71	1-Isopropyl-4, 7-dimethyl-1, 2, 3, 4, 5, 6-hexahydronaphthalene	26.89	C_15_H_24_	16729-00-3	59.31	1649	0.12
72	(4aR-trans)-Decahydro-4a-methyl-1-methylene-7-(1-methylethylidene)-naphthalene	27.09	C_15_H_24_	515-17-3	80.30	1662	0.62
73	trans-Valerenyl acetate	27.14	C_17_H_26_O_2_	101527-74-6	72.98	1665	0.21
74	(E)-2-((8R, 8aS)-8, 8a-Dimethyl-3, 4, 6, 7, 8, 8a-hexahydronaphthalen-2 (1H)-ylidene) propyl formate	27.41	C_16_H_24_O_2_	352457-47-7	54.29	1679	0.72
75	cis-α-Copaene-8-ol	27.79	C_15_H_24_O	58569-25-8	55.35	1694	0.29
76	Heptadecane	27.98	C_17_H_36_	629-78-7	68.57	1702	0.19
77	(E)-1, 3, 3-trimethyl-2-(3-methyl-2-methylene-3-butenylidene)-cyclohexanol	28.27	C_15_H_24_O	69296-93-1	50.18	1716	0.14

* The relative peak area normalization content obtained from the TIC chromatogram.

## Supplementary Materials

The following are available online at https://www.mdpi.com/2304-8158/8/6/205/s1, Figure S1: Comparison of the peak numbers obtained from different conditions: (a) different equilibrium temperatures; (b) different equilibrium time, Figure S2: The comparison of the total ion chromatograms of ICS samples: (a) the ICS essential oil; (b) the ICS powder in NADES-2 containing 15% water.
