# Supplementary material for: Headspace Gas Chromatography-Mass Spectrometry for Volatile Components Analysis in Ipomoea Cairica (L.) Sweet Leaves: Natural Deep Eutectic Solvents as Green Extraction and Dilution Matrix
Source: Foods. 2019 Jun 11;8(6):205. doi: 10.3390/foods8060205 (PMC6617084; doi:10.3390/foods8060205)
Supplement: Supplementary file 1 [file foods-08-00205-s001.pdf]

Headspace gas chromatography-mass spectrometry for volatile components analysis in *Ipomoea cairica* (L.) *Sweet* leaves: Natural deep eutectic solvents as green extraction and dilution matrix

Wei Zhang and Xianrui Liang *

Collaborative Innovation Center of Yangtze River Delta Region Green Pharmaceuticals, College of Pharmaceutical Sciences, Zhejiang University of Technology, Hangzhou 310014, China; liangxrvicky@zjut.edu.cn

* Correspondence: liangxrvicky@zjut.edu.cn; Tel.: +86-571-8832-0420

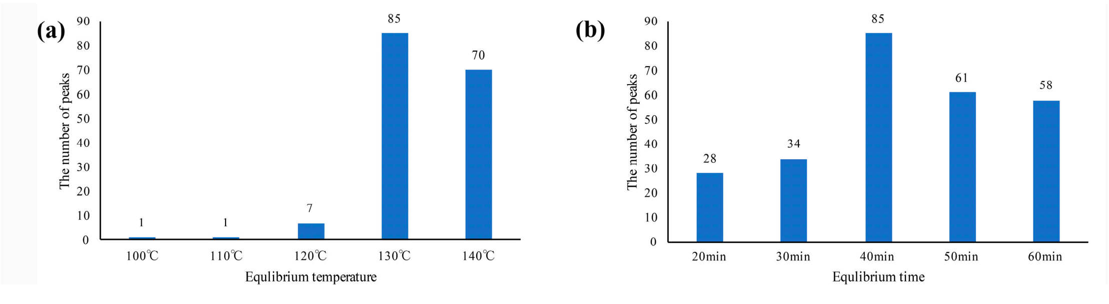

Figure S1. Comparison of the peak numbers obtained from different conditions: (a) different equilibrium temperatures; (b) different equilibrium time.

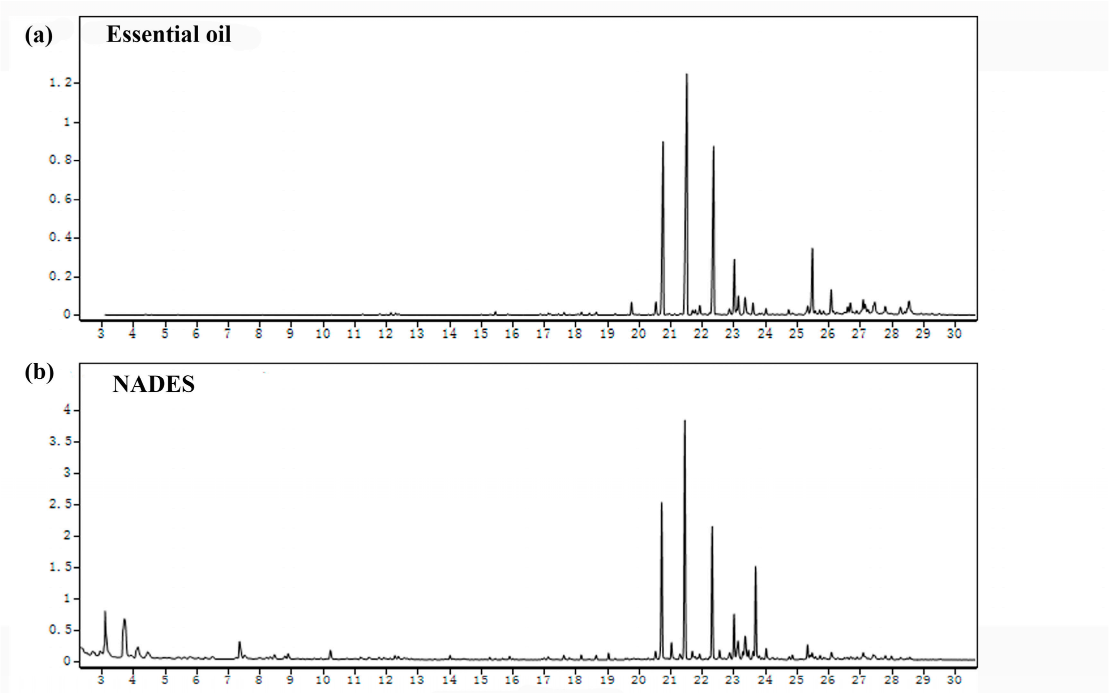

Figure S2. The comparison of the total ion chromatograms of *ICS* samples: (a) the *ICS* essential oil ; (b) the *ICS* powder in NADES -2 containing 15% water.
